# A Predictive Analysis of Heart Rates Using Machine Learning Techniques

**DOI:** 10.3390/ijerph19042417

**Published:** 2022-02-19

**Authors:** Matthew Oyeleye, Tianhua Chen, Sofya Titarenko, Grigoris Antoniou

**Affiliations:** Department of Computer Science, School of Computing and Engineering, University of Huddersfield, Huddersfield HD1 3DH, UK; matthew.oyeleye@hud.ac.uk (M.O.); s.titarenko@hud.ac.uk (S.T.); g.antoniou@hud.ac.uk (G.A.)

**Keywords:** heart rate, accelerometer, time series, data analytics, machine learning

## Abstract

Heart disease, caused by low heart rate, is one of the most significant causes of mortality in the world today. Therefore, it is critical to monitor heart health by identifying the deviation in the heart rate very early, which makes it easier to detect and manage the heart’s function irregularities at a very early stage. The fast-growing use of advanced technology such as the Internet of Things (IoT), wearable monitoring systems and artificial intelligence (AI) in the healthcare systems has continued to play a vital role in the analysis of huge amounts of health-based data for early and accurate disease detection and diagnosis for personalized treatment and prognosis evaluation. It is then important to analyze the effectiveness of using data analytics and machine learning to monitor and predict heart rates using wearable device (accelerometer)-generated data. Hence, in this study, we explored a number of powerful data-driven models including the autoregressive integrated moving average (ARIMA) model, linear regression, support vector regression (SVR), k-nearest neighbor (KNN) regressor, decision tree regressor, random forest regressor and long short-term memory (LSTM) recurrent neural network algorithm for the analysis of accelerometer data to make future HR predictions from the accelerometer’s univariant HR time-series data from healthy people. The performances of the models were evaluated under different durations. Evaluated on a very recently created data set, our experimental results demonstrate the effectiveness of using an ARIMA model with a walk-forward validation and linear regression for predicting heart rate under all durations and other models for durations longer than 1 min. The results of this study show that employing these data analytics techniques can be used to predict future HR more accurately using accelerometers.

## 1. Introduction

According to the World Health Organization (WHO), heart disease (HD), also known cardiovascular disease (CVD), is one of the major causes of mortality in the world today [1]. It reported that 17.9 million people were estimated to have died from CVDs in 2019, accounting for 32% of all global deaths. Heart disease describes a series of conditions that affect the heart, which in turn affects the heart to pump blood around the body normally [2]. However, there is no way to track cardiovascular or heart disease without considering the heart rate (HR), which is one of the important measures of heart health. The HR is the number of times the heart’s chambers contract (squeeze) and relax to pump blood within a specified period (i.e., minute) and at rest, a normal heart beats approximately 60–80 times per minute [2]. The heart rate, however, is affected by the activities a human engages in and in turn, the heart rate data are nonstationary in nature, which are unpredictable and cannot be modelled or forecasted [3,4]. This may be complicated by unpredictability attributes and other behavioral risk factors such as tobacco use, unhealthy diet and obesity, physical inactivity and harmful use of alcohol, which contribute to worse wellbeing and may even double the death risk of a CVD patient [4,5]. It is then important to detect cardiovascular disease as early as possible.

Recent advancement in artificial intelligence is bringing a paradigm shift to healthcare, ranging from early disease detection and diagnosis, to personalized treatment and prognosis evaluation [6,7,8,9,10]. The ongoing revolution in health and clinical examination procedures has continued to witness improvement with the increasing rates of wearable sensors [11]. For example, the health monitoring system is currently monitoring a patient’s cardiovascular conditions at home in order to provide appropriate recommendations to both patients and the medical consultants [12]. The low cost and non-invasive feature of the wearable devices has made it possible to record large quantities of physiological data, track medications, follow the recovery of post-op patients and track sleep, which in turn, provides real-time health monitoring of vital statistics, providing more timely data for analysis and earlier detection of disease or the risk of major health events.

This huge contribution to the fast-growing IoT and wearable monitoring system in the healthcare space has played a vital role in early detection of a high heart rate to prevent the risk of cardiovascular disease progression. Early detection and diagnosis of cardiovascular disease are very important because it is easier to manage and treat at early stages of the disease [1,4].

Several research works have used various techniques such as statistical models, machine learning models and historical data to measure various risks factors of several diseases. Recently, new novel mechanical elements have been used as wearable sensors and actuators due to their incredibly small sizes. Accelerometers are sensors that are used to accurately monitor human activity by measuring external forces along a reference axis. Accelerometers can be tools to monitor heart rates [13]; they generate time-series streaming heart rate data that can be processed on a row-by-row basis by time progression. In recent research, [14] used an accelerometer to monitor several subjects and their 24 h HR and shared their collection of raw data including several other 24 h continuous psycho-physiological information that enables investigation of possible relationships between the physical and psychological characteristics of people in daily life. Further, the combination of these data enables the development of tools that can predict the users’ well-being. However, the HR time-series dataset provided by the study needs to be analyzed in a consecutive and incremental way using a sliding time window approach. In working towards employing data analytics and machine learning to analyze the effectiveness of using accelerometer data to monitor and predict heart rates, we explored several data analytics techniques in this study for the analysis of accelerometer data to make future HR predictions from the accelerometer’s univariant HR time-series dataset. 

Over the last few decades, there has been much research directed at understanding and predicting the future from time series data. In the literature, several linear approaches have been proposed for time series forecasting. Autoregressive integrated moving average (ARIMA) models have gained popularity as linear models over the past three decades [15] and hence, have been widely applied to construct more accurate hybrid models in time series forecasting. ARIMA models have been applied for forecasting in many fields such as health, social, economic, engineering, foreign exchange and stock problems. A study by [16] used ARIMA to perform a spatial prediction of the COVID-19 epidemic to forecast the epidemiologic pattern in India. Further, ARIMA was used to forecast coronavirus disease in Indonesia in a study by [17]. An investigation on the effect of post-traumatic stress disorder (PTSD) on various factors including heart rate by [18] employed ARIMA models to analyze the heart rate data. An ARIMA model was used to capture the trend of pulse production in India by [19] to predict pulse production from 2020 to 2029 to bridge the gap between the supply and demand. Two time series models were employed to estimate the growth rate of glioblastoma in response to ionizing radiotherapy treatment in a comparative study presented by [20]. Their study showed that ARIMA performed better based on the mean square error (MSE) and MAPE values obtained than the Holt method. Ref. [21] applied an ARIMA time series model to forecast the future gold price in India to mitigate the risk in gold purchases. A study presented by [22] proposed a novel approach to improve an ARIMA model by applying a mean estimation error for time series forecasting. A novel hybridization of artificial neural networks (ANNs) and an ARIMA model was proposed by [15] to overcome limitations of ANNs. Their model produced more general and more accurate forecasting than traditional hybrid ARIMA–ANNs models. However, ARIMA models perform best when the time series is stationary and the data are free from missing values that may be imputed through advanced interpolation techniques [23,24]. Due to the linearized nature of ARIMA, which may not capture nonlinear behavior [25], it is unreasonable to assume that a particular realization of a given time series is generated by a linear process. This limitation has led to the exploration of alternatives to statistical linear models: machine learning and deep learning.

Many studies have been conducted using machine learning and deep learning to examine time series data. A study by [26] explored the applicability of machine learning and the advantages of recurrent neural networks (RNNs) for pore-water pressure (PWP) time-series prediction. A comparative investigation between different deep learning models such as LSTM, BI-LSTM and CNN, using univariate and multivariate time-series data, was conducted by [27] for forecasting blood pressure and heart rate. The models were used to predict blood pressure (BP) 30 min in advance and HR 30 min in advance as univariates and to predict BP and HR as multivariates. In work by [28], a novel hybrid machine learning technique was proposed to improve the accuracy in the prediction of cardiovascular disease. Their prediction model produced an enhanced performance level with an accuracy level of 88.7% using a hybrid random forest with a linear model (HRFLM). A real-time prediction system for heart rate was proposed by [4] using deep learning and stream processing platforms using heart rate time-series dataset extracted from Medical Information Mart for Intensive Care (MIMIC-II). Their proposed system consists of two phases, namely, an offline phase and an online phase. Different deep learning forecasting techniques were used to find the lowest mean square error for the offline phase. The best developed model from the offline phase was used to predict the heart rate in advance from the online phase. In a telehealth system architecture developed by [29] for monitoring the cardiovascular risk, a fuzzy inference system (FIS) was employed to predict the level of cardiovascular risk from vital parameters related to cardiovascular diseases such as heart rate, respiration rate, blood oxygen saturation and color of lips that were collected through a contact-less smart object. A machine learning approach was proposed to improve the accuracy of HR detection in naturalistic measurements in study by [30]. A four-layer deep neural network, two CNN layers and two LSTM layers, was used by [31] to model and predict heart rate. The proposed network was evaluated on the TROIKA dataset with 22 PPG records collected during various physical activities. The proposed system achieved an improved mean absolute error accuracy for heart rate prediction. A novel deep learning framework was developed by [32] for real-time heart rate estimation from facial video captured by an RGB camera. [33] proposed the use of an LSTM deep learning model for initial diagnosis of heart failure (HF). Their proposed model was compared with other baseline models such as multilayer perceptron (MLP), logistic regression, k-nearest neighbor (KNN) and support vector machine (SVM). The results show that the proposed model achieved the best accuracy compared to other algorithms.

However, none of the methods is a universal model that is suitable for all circumstances. The approximation of ARIMA models to complex nonlinear problems as well as machine learning to model linear problems may be totally inappropriate, as well as for problems that consist of both linear and nonlinear correlation structures. Using hybrid models or combining several models has become a common practice in order to overcome the limitations of components models and improve the forecasting accuracy [15]. A study presented by [34] used two approaches for energy consumption forecast: an autoregressive integrated moving average (ARIMA) model and a non-linear autoregressive neural network (NAR) model.

Hence, the limitations and the inapplicability of using a specific method for solving time-series prediction problems shows a need to explore the effectiveness of these popular forecasting techniques in cardiovascular disease prediction using a 24 h accelerometer-generated HR time-series recordings. To the best of our knowledge, none of the existing studies on heart rate prediction used the ARIMA model for predicting future heart rates. For this reason, in this paper, we employed the ARIMA model, regression models and a deep learning model for predicting heart rates. The data analytics methods included an autoregressive integrated moving average (ARIMA) model, linear regression, support vector regression (SVR), k-nearest neighbor (KNN) regressor, decision tree regressor, random forest regressor and a long short-term memory (LSTM) recurrent neural network algorithm. We compared the performances of these models by evaluating the root mean squared error (RMSE) and calculating the scatter index (SI) of each model against the different sliding windows.

Our experimental results prove that the ARIMA model can better perform in predicting future heart rates from univariant heart rate time-series data than machine and deep learning models. Thus, our findings demonstrated that ARIMA is a better model for predicting future heart rates more accurately.

## 2. Materials and Methods

### 2.1. Data

In this study, we used the Multilevel Monitoring of Activity and Sleep in Healthy people (MMASH) dataset [14] providing 24 h of continuous inter-beat interval data (IBI), triaxial accelerometer data, sleep quality, physical activity and psychological characteristics (i.e., anxiety status, stress events and emotions) for 22 healthy young males (age = 27.29 ± 4.21 years; height = 179.91 ± 8.22 cm; weight = 75.05 ± 12.79 kg). Participants’ anthropomorphic characteristics (i.e., age, height, and weight) were recorded at the start of the data recording. Moreover, participants filled in questionnaires that provide information about their psychological status, i.e., chronotype, anxiety status and sleep quality. During the 24-h data recording, participants wore two devices that continuously recorded heart response (Polar H7 heart rate monitor-Polar Electro Inc., Bethpage, NY, USA) and Actigraph data (ACTi Graph wGT3X-BT-ACTi Graph LLC, Pensacola, FL, USA). Moreover, the perceived moods were recorded at different times of the day, and the daily stress was provided before sleeping in order to summarize the individual’s stressful events of the day. Finally, twice a day (i.e., before going to bed and when they woke up), the subjects collected saliva samples at home in appropriate vials in order to assess the melatonin and cresol saliva concentration. More details about the experimental setup of the MMASH dataset are provided in the data descriptor paper [14].

In this study, we used the IBI and Actigraph data that were continuously recorded over 24 h on 22 healthy young males using a Polar H7 chest strap (Polar Electro Inc., Bethpage, NY, USA) and Actigraph (Actigraph wGT3X-BT-Actigraph LLC, Pensacola, FL, USA), respectively. During the test, participants wore two devices continuously for 24 h: (between 9:00 a.m. and 9:00 p.m. on the next day) and were instructed to wear the chest straps during both the day (during physical activities too) and at night. The heart rate time series (univariate dataset) from Actigraph dataset was recorded on a second-by-second basis for one subject.

### 2.2. Data Pre-Processing

To detect ectopic beats (i.e., disturbance of the cardiac rhythm frequently related to the electrical conduction system of the heart) or missing values induced by motion artifacts from the Actigraph data, we used the Python hrv-analysis library (https://pypi.org/project/hrv-analysis, accessed on 6 February 2021) to reconstruct the RR-intervals from the inter-beat-interval (IBI) dataset to obtain the maximum and minimum heart rate that was used to filter out the outliers from the Actigraph dataset.

Due to the nonstationarity of the HR time-series dataset, we had to transform it into a stationary dataset to be fitted with the prediction model using a transformation method called differencing, which is described by Equation (1) below. The differencing method’s function was used to remove the series dependence on time by subtracting the values of successive HRs of a certain period from the last values of the time series to eliminate varying means.

(1) difference(t)=observation(t)−observation(t−1)

### 2.3. Autoregressive Integrated Moving Average (ARIMA) Model

The ARIMA model is effective in capturing a suite of different standard temporal structures in time series data. However, configuring the ARIMA model with the best tuning parameters p, q and q (the lag order, degree of differencing and the order of the moving average, respectively) that require estimation by iterative trial and error can be challenging [35]. Fitting of the ARIMA model followed the Box–Jenkis methodology classical approach [36]. To determine the best fit configuration hyperparameter of the ARIMA model, the model was tuned with an automated GridSearch algorithm that evaluates ARIMA models on different combinations of model hyperparameters and obtains the best fit tunning configuration. We specified a grid of p, d and q of the ARIMA parameters to iterate between the ranges of 0–10, 0–3 and 0–3, respectively. The GridSearch automates the process of training and evaluating ARIMA models on the different combinations of model hyperparameters by keeping the track of the lowest error score observed and the configuration that caused it as each time step of the test set is iterated.

Cross-validation is one of the most widely used methods to evaluate a model performance, as it is very important to prevent model overfitting. However, cross-validation is trivial in the case of time series [37]. There is a temporal dependency between observations that must be preserved during testing. To cross-validate the ARIMA model, we used a walk-forward validation that uses a rolling forecast technique that uses a small subset of data for training purpose, predicts the later data points and then checks the accuracy of the predicted data points. A new ARIMA model was recreated after each new observation was received, using the same predicted data points as part of the next training dataset, and subsequent data points were predicted. We manually kept track of all observations in a list called history that was seeded with the training data and to which new observations were appended after each iteration.

### 2.4. Machine Learning Techniques

The machine learning models learn the association function between pairs of input and output sequences known as input and output variables, denoted by (X) and (y), respectively, to make predictions. Therefore, to make predictions from the univariant HR time-series datasets, we reframed the time-series into a supervised learning problem by converting the sequenced HR time-series observation window (X) and target window (y). We configured this by using the observation from the last time step (t−1) as the input and the observation at the current time step (t) as the output.

#### 2.4.1. Linear Regression

Linear regression is probably one of the most important and widely used regression techniques. It is among the simplest regression methods. One of its main advantages is the ease of interpreting results [38]. Linear regression fits a linear model with coefficients w = (w1, …, wp) to minimize the residual sum of squares between the observed targets in the dataset and the targets predicted by the linear approximation [39]. We trained and fit the linear regression model with different data sizes according to the sliding window duration, predicted future HRs and calculated the error scores for each experiment.

#### 2.4.2. Support Vector Regression (SVR)

Support vector regression is a type of support vector machine that supports linear and non-linear regression. It is used to predict discrete values. However, the basic idea behind SVR is to find the best fit line, i.e., to make sure that the errors do not exceed the threshold. 

To achieve this in our experiment, we fine-tuned the SVR kernel and regularization parameter C hyperparameters to linear and 1, respectively. The model was trained with different training data sizes according to the sliding window duration and was fit to make predictions in each experiment.

#### 2.4.3. K-Nearest Neighbor (KNN) Regressor

The K nearest neighbor is a simple algorithm that stores all available cases and predicts the numerical target based on a similarity measure (e.g., distance functions). A simple implementation of KNN regression is to calculate the average of the numerical target of the K nearest neighbors. However, the KNN regressor algorithm performs best when we have a minimized error, i.e., error calculation for our training and validation sets. This is highly determined by the optimum value of k. 

To determine the optimum value of k, we applied the GridSearch algorithm to the KNN regressor algorithm. The GridSearch algorithm automatically helps to find the best value of K after running a certain number of iterations on the KNN regressor’s model. We configured the GridSearch algorithm to evaluate the KNN regressor, setting the KNN regressor’s n_neighbor parameter between the range of 1–9 for 10 iterations. We obtained the best n_ neighbor value and used it to configure the KNN regressor model for each sliding window duration experiment.

#### 2.4.4. Decision Tree Regressor

A decision tree is a supervised learning algorithm that has a graphical representation of all the possible solutions. It starts from the root node and branches to find the solution based on some conditions. A decision tree model is also a good model for both regression and classification problems that uses a binary rule to learn the relationship between the data and the target variable and for prediction. It normally uses the mean squared error (MSE) to decide to split a node in two or more sub-nodes. Often, the model may be underfitted or overfitted to the data, which in most cases is detrimental to the model’s performance when new data are introduced. 

Thus, to prevent our decision tree regressor model from being underfitted or overfitted when training, we set constraints on tree size by fine-tuning hyperparameters. We iterated the depth with a range of 2–10. For each depth step, we set the decision tree regressor model max_depth hyperparameter value to the current depth step, fit the model with our training data and calculated the error score. We kept track of the lowest error score observed and the depth that caused it.

#### 2.4.5. Random Forest Regressor

The random forest regressor is also a supervised learning algorithm with a collection of decision trees, which are less prone to overfitting and perform better than a single optimized tree [40]. It runs predictions on each individual tree and then averages their predictions to create the final prediction, thus making it quite slow to create predictions once trained, but it can be fast to train. However, the random forest regressor model may be overfitted, which makes it performs well for the training set but poorly for the test set, which may make it not applicable to new problems.

To overcome the overfitting problem, we also employed the GridSearch algorithm to fine-tune the hyperparameters. The approach allowed us to explicitly specify the combination of parameters to be tested. We set the estimator, param_grid, cv, n_jobs and verbose to randomforestregressor (), {’bootstrap’: [True], ’max_depth’: [80,90,100,110], ’max_features’: [2,3],’min_samples_leaf’: [3–5], ’min_samples_split’: [8,10,12], ’n_estimators’: [100,200,300,1000]}, 3, −1 and 2, respectively. The best_param from the GridSearch algorithm result was used to fit our random forest regressor model, and we evaluated the model.

#### 2.4.6. LSTM Deep Learning Model

Recently, deep learning models have become a promising tool for time series forecasting because of their strength in the automatic learning of temporal dependence and the automatic handling of temporal structures such as trends and seasonality as well as their ability to automatically learn arbitrary complex mappings from inputs to outputs and to support multiple inputs and outputs [41]. However, there is a deep learning model termed a convolutional neural network (CNN). CNNs are neural networks and deep learning models that have the capability to learn and automatically extract features from raw input data and can be applied to time series forecasting problems but were designed to efficiently handle image data. Long short-term memory networks (LSTMs) are recurrent neural networks (RNNs) and deep learning models that add the explicit handling of order between observations when learning a mapping function from inputs to outputs, which is not offered by CNNs. It has capability to add support for input sequence data as well learned temporal dependence, i.e., learns mapping from inputs to outputs and learns what context from the input sequence is useful for the mapping and can dynamically change this context as needed.

To make the experiment fair, LSTM models usually work with a scaled coefficient (min. and max.) value within their activation function ranges; the output values range between −1 and 1. We normalized the dataset to the range [−1, 1] using the Python library function MinMaxScaler class, i.e., MinMaxScaler (feature_range = (−1, 1)) [42]. To evaluate the model, the predictions were transformed back to the original scale so that the result could be interpreted and a comparable error score could be calculated. To invert scaling, that is, reverse the scaled data back to the original values, we also used another function from the Python library. The LSTM model consists of input sequences in terms of numbers of lags, hidden layer(s) and an output layer including a dense layer that produces the output. Since our HR time-series is a univariate series, the number of features is one, for one variable. Our LSTM networks was stacked with two hidden layers and an output layer using memory between batches, which allowed us to make predictions for different sliding window sizes. The model was fit using the Adam optimizer [43] and was optimized using the mean squared error, or ‘mse‘ loss function.

### 2.5. Data Splitting

The Actigraph HR time-series data were split into 67% as a training set and 33% as a testing set for each sliding window, following recent works of [44] and [45]. All models were trained and optimized by the training set and evaluated by the testing test.

### 2.6. Model Evaluation

To evaluate the models, the 22 participants’ Actigraph datasets were used; 30 s, 1 min, 5 min, 10 min, 15 min, 30 min and 1 h sliding window data were extracted for each participant and split using the proportion mentioned above for training and testing sets. Precision and recall have been the standard metrics for evaluating time series classification algorithms [46], which are also alternatives to calculate the classification model accuracy [47]. Since our study focused on time-series regression problems, our model performance was measured using various time-series regression model metrics as used by [4] and suggested by [48]. The models were trained and used to make predictions for each sliding window, and the average values of the mean average error, mean square error and root mean square error were calculated.

Since the root mean square error (RMSE) value is directly proportional to the unit of the predicted values, it has to be understood that we have to take a look at the importance of the RMSE in comparison with the predicted values. To know if it is good or bad, the scatter index (SI), which is simply the RMSE divided by the average value of the observed value, was computed. SI = (RMSE/average observed value) * 100%. If SI < 10% is a good model, SI < 5% is a very good model. Conversely, a model of prediction has to have a high R2 (closer to 1), which shows that the regression line fits the data well and the model performance is good, and SI less than 30% if we consider annual data and 10% if we consider hourly or monthly data [49]. Each model’s performance was evaluated using RMSE and SI:

(2) $$RMSE=\sqrt{\left(\frac{1}{n}\right)\overset{n}{\underset{i=1}{\sum }}{\left(y_{i}^{obs}-y_{i}^{pred}\right)}^{2}}$$

(3) $$SI=\left(RMSE-\left(\frac{1}{n}\right)\overset{n}{\underset{i=1}{\sum }}y_{i}^{obs}\right)\times 100$$

## 3. Results

Our study used the autoregressive integrated moving average (ARIMA) model, linear regression, support vector regression (SVR), k-nearest neighbor (KNN) regressor, decision tree regressor, random forest regressor and long short-term memory (LSTM) recurrent neural network algorithm to predict future HR from a univariant HR time-series data obtained from an Actigraph dataset of 22 healthy subjects. Each model was evaluated using RMSE and SI of different sliding windows (30 secs, 1 min, 3 min, 5 min, 10 min, 15 min, 30 min and 1 h), and the average RMSE and SI for all subjects were computed for each sliding window.

We experimentally demonstrate the model performance using a sliding window of 30 min for prediction. The ARIMA and SVR models had the best SI scores of 0.00% and 0.29%, respectively, while the KNN regressor and LSTM performed the worst, with SI scores of 41.36% and 34.15%, respectively, as shown in Table 1.

In the 1 min sliding window experiment, the ARIMA and SVR models had the best SI scores of 0.00% and 0.29%, respectively, while the KNN regressor and LSTM models performed the worst, with SI scores of 41.36% and 34.15%, respectively, as shown in Table 2 below.

The ARIMA model and linear regression models performed best for the 3 min sliding window, with SI scores of 1.38% and 1.76%, respectively. KNN and LSTM showed fair performance with SI scores of 3.62% and 3.31%, respectively, as shown in Table 3 below.

Evaluation of the models for the 5 min sliding window showed that the ARIMA model and linear regression model had the best performance, with SI scores of 1.57% and 1.80%, respectively, and the models with a fair performance were the LSTM and SVR models, with SI scores of 3.27% and 3.21%, respectively, as shown in Table 4.

In the 10 min sliding window experiment, the ARIMA model and the linear regression model again performed the best, with SI values of 1.36% and 1.38%, and the SVR and LSTM models had a fair performance, with SI values of 2.68% and 2.36%, respectively, as shown in Table 5 below.

For the 15 min sliding window experiment, the ARIMA model and the linear regression showed the best performance, with SI values of 1.33% and 1.44%, while the KNN and random forest model performed poorly, with SI values of 5.87% and 5.25%, respectively, as shown in Table 6.

When the experiment was carried out on a 30 min sliding windows, the results showed that the ARIMA model and the linear regression also performed the best, with SI values of 1.64% and 1.67%, and the LSTM and SVR models had a fair performance, with SI values of 2.33% and 2.17%, respectively, as shown in Table 7 below.

Finally, the models were also evaluated for the 1 h HR recording sliding windows. The logistic regression and the ARIMA models also had the best performances, with SI scores of 1.63% and 1.17%, respectively while the LSTM and the KNN models had a fair performance, with SI scores of 3.04% and 2.10%, respectively, as shown in Table 8.

## 4. Discussion

In our study, we used the 24 h accelerometer-generated HR time-series research data provided by [14] for prediction. This may not be an efficient way to capture HR data [50], compared to more accurate HR data recorded by an electrocardiogram, which are not applicable and suitable for everyday use [31]. However, the research dataset also captured the IBI recordings that we reconstructed to filter out ectopic heart beats from the accelerometer data, thus producing more reliable and accurate data.

The results of the study showed a very close evaluation score for the 30 s and 1 min sliding windows, which indicated very few HR fluctuations within the duration of 30 s; therefore, using 30 s of HR recording is not sufficient to make predictions in the case where a high degree of fluctuations in the HR is expected to occur in the future.

**Figure 1:**
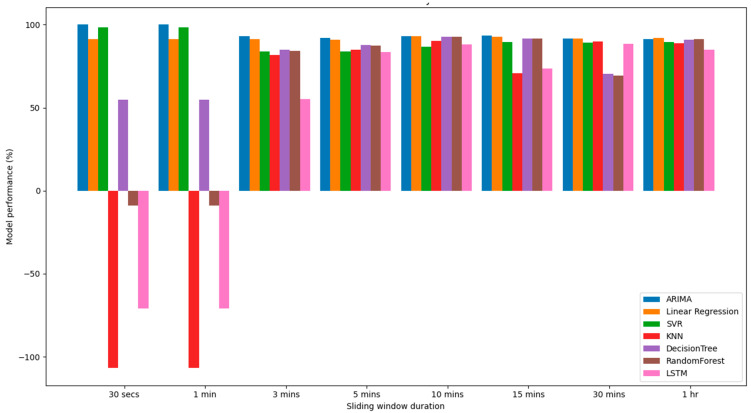
Pictorial representation of the models’ SI performances.

Model performance with SI scores less than 5% is considered to be a very good model to make predictions for our second-to-second HR time-series data, i.e., the closer the model performance is to 0%, the closer the performance is to 100%. To visualize this, we computed each model’s performance on a scale of 100% against each sliding window as shown in Figure 1 below. It was observed that some model performances were on the negative scale, which indicates how far their SI values are from 0%.

Further, the study also showed that the ARIMA and linear regression models performed the best in all experiments, and the KNN, LSTM and random forest regressor models performed very poorly; the decision tree regressor model had average performance for the 30 s and 1 min windows. The SVR model also performed better in the first two experiments, i.e., the 30 s and 1-min windows; however, similar to the other models such as KNN, decision tree regressor, random forest regressor and the LSTM, the performance for other experimental sliding windows was relatively better but unstable. However, our results also indicated that the RMSE and SI were the best in ultra-short (i.e., between 30 s and 4 min) sliding window durations. This is due the fact that there is a decrease in bias towards the HR as a result of limited HR fluctuations, which is also a good parameter to measure the heart rate variability (HRV) [51].

A comparison of the results of each sliding window in this study to the results of the corresponding sliding window obtained in recent studies by [4] and [30] shows that some of the techniques we explored performed better than the techniques used in their approaches.

## 5. Conclusions

This study has addressed the use of machine learning to predict HR using 24 h univariant HR time-series data generated by an accelerometer, which can be used to detect early HR risks and to monitor patients with heart disease. We used the autoregressive integrated moving average (ARIMA) model, linear regression, support vector regression (SVR), k-nearest neighbor (KNN) regressor, decision tree regressor, random forest regressor and long short-term memory (LSTM) recurrent neural network algorithm to make predictions from the HR time-series. Each model was evaluated using RMSE and SI against different sliding windows of 30 s, 1 min, 3 min, 5 min, 10 min, 15 min, 30 min and 1 h. Our results showed that the ARIMA model with a walk-forward validation and linear regression was the best to make future HR predictions to track HR-related risks over time with any given HR recording durations. The KNN, LSTM and random forest regressor models are not good models for prediction from an HR recording duration of 1 min or shorter, and the KNN, LSTM and random forest regressor models and other models such as decision tree regressor and SVR can also be used to make better HR predictions from longer recording windows.

The results of this study show that the research data provided by [14] are sufficient, reliable and can be explored using several data analytics techniques to predict future HR using an accelerometer.

## Figures and Tables

**Table 1:** The results of the models for the 30 s sliding window.

	Model	Mean Average Error	Mean Square Error	Root Mean Square Error	Scattered Index
**30 s**	ARIMA	0	0	0	0
	Linear Regression	3.12	9.75	3.12	1.76
	SVR	0.51	0.26	0.51	0.29
	KNN	73.2	5358.24	73.2	41.36
	Decision Tree	16	256	16	9.04
	Random Forest	38.5	1482.1	38.5	21.75
	LSTM	60.45	3653.93	60.45	34.15

**Table 2:** The results of the models for the 1 min sliding window.

	Model	Mean Average Error	Mean Square Error	Root Mean Square Error	Scattered Index
**1 min**	ARIMA	0	0	0	0
	Linear Regression	3.12	9.75	3.12	1.76
	SVR	0.51	0.26	0.51	0.29
	KNN	73.2	5358.24	73.2	41.36
	Decision Tree	16	256	16	9.04
	Random Forest	38.5	1482.1	38.5	21.75
	LSTM	60.45	3653.93	60.45	34.15

**Table 3:** The results of the models for the 3 min sliding window.

	Model	Mean Average Error	Mean Square Error	Root Mean Square Error	Scattered Index
**3 min**	ARIMA	0.9	1.63	1.28	1.38
	Linear Regression	1.41	2.7	1.64	1.76
	SVR	2.58	8.93	2.99	3.2
	KNN	3.07	11.38	3.37	3.62
	Decision Tree	2.52	7.86	2.8	3
	Random Forest	2.67	8.69	2.95	3.16
	LSTM	2.35	9.52	3.08	3.31

**Table 4:** The results of the models for the 5 min sliding window.

	Model	Mean Average Error	Mean Square Error	Root Mean Square Error	Scattered Index
**5 min**	ARIMA	0.87	2.08	1.44	1.57
	Linear Regression	1.18	2.74	1.65	1.8
	SVR	2.66	8.74	2.96	3.21
	KNN	2.17	7.7	2.78	3.01
	Decision Tree	1.76	5.07	2.25	2.45
	Random Forest	1.79	5.52	2.35	2.55
	LSTM	2.54	9.05	3.01	3.27

**Table 5:** The results of the models for the 10 min sliding window.

	Model	Mean Average Error	Mean Square Error	Root Mean Square Error	Scattered Index
**10 min**	ARIMA	0.82	1.48	1.22	1.36
	Linear Regression	0.93	1.5	1.23	1.38
	SVR	2.08	5.7	2.39	2.68
	KNN	1.42	3.11	1.76	1.98
	Decision Tree	1.04	1.72	1.31	1.47
	Random Forest	0.98	1.61	1.27	1.42
	LSTM	1.75	4.42	2.1	2.36

**Table 6:** The results of the models for the 15 min sliding window.

	Model	Mean Average Error	Mean Square Error	Root Mean Square Error	Scattered Index
**15 min**	ARIMA	0.72	1.19	1.09	1.33
	Linear Regression	0.93	1.4	1.18	1.44
	SVR	1.44	2.99	1.73	2.1
	KNN	3.86	23.32	4.83	5.87
	Decision Tree	2.69	12.22	3.5	4.25
	Random Forest	3.36	18.63	4.32	5.25
	LSTM	2.74	9.56	3.09	3.76

**Table 7:** The results of the models for the 30 min sliding window.

	Model	Mean Average Error	Mean Square Error	Root Mean Square Error	Scattered Index
**30 min**	ARIMA	0.88	1.97	1.4	1.64
	Linear Regression	0.99	2.05	1.43	1.67
	SVR	1.44	3.48	1.87	2.17
	KNN	1.3	3.11	1.76	2.05
	Decision Tree	1.03	2.07	1.44	1.67
	Random Forest	1.03	2	1.42	1.65
	LSTM	1.63	4.01	2	2.33

**Table 8:** The results of the models for the 1 h sliding window.

	Model	Mean Average Error	Mean Square Error	Root Mean Square Error	Scattered Index
**1 h**	ARIMA	0.93	2.34	1.53	1.71
	Linear Regression	0.97	2.13	1.46	1.63
	SVR	1.37	3.53	1.88	2.1
	KNN	1.42	3.99	2	2.23
	Decision Tree	1.1	2.64	1.63	1.82
	Random Forest	1.07	2.5	1.58	1.77
	LSTM	2.15	7.38	2.72	3.04

## Data Availability

The dataset analyzed in this study was released under the Open Database License (ODbL) v1.0 and is publicly available on PhysioNet. The data can be found here: https://physionet.org/content/mmash/1.0.0/ (accessed on 19 June 2020).
